# Inhibition of biofilm formation and preformed biofilm in *Acinetobacter baumannii* by resveratrol, chlorhexidine and benzalkonium: modulation of efflux pump activity

**DOI:** 10.3389/fmicb.2024.1494772

**Published:** 2024-12-16

**Authors:** Antonella Migliaccio, Maria Stabile, Maria Triassi, Emmanuelle Dé, Eliana De Gregorio, Raffaele Zarrilli

**Affiliations:** ^1^Department of Public Health, University of Naples Federico II, Naples, Italy; ^2^Department of Molecular Medicine and Medical Biotechnology, University of Naples Federico II, Naples, Italy; ^3^University of Rouen Normandie, National Institute of Applied Sciences (INSA) Rouen Normandie, Centre National de la Recherche Science (CNRS), Lab. Polymers, Biopolymers, Surfaces (PBS), Unité Mixte de Recherche, Rouen, France

**Keywords:** *Acinetobacter baumannii*, biocides, resveratrol, biofilm growth, preformed biofilm, efflux pumps

## Abstract

**Introduction:**

The persistence of *Acinetobacter baumannii* in the contaminated environment is sustained by tolerance to biocides and ability to growth as biofilm. The aim of the study was to analyze the susceptibility of *A. baumannii* biofilms to chlorhexidine (CHX) and benzalkonium (BZK) biocides and the ability of natural monomeric stilbenoid resveratrol (RV) to modulate the phenomenon.

**Methods:**

Biofilm formation and preformed biofilm were tested by Crystal violet and tetrazolium salt reduction assay, respectively. Analysis of efflux pump (EP) expression during biofilm growth was performed by Real-time RT-PCR assays.

**Results:**

CHX and BZK at ¼ and ½ MICs alone or in combination inhibited biofilm growth of *A. baumannii* ATCC 19606, 4190, and 3909 strains. RV at 32 mg/L and CHX and BZK at ¼ or ½ MICs showed a synergistic effect and completely inhibited biofilm formation in all *A. baumannii* strains. Similarly, RV at 32 mg/L and CHX and BZK at ½ MIC significantly inhibited air-liquid biofilm formation of *A. baumannii* ATCC 19606, 4190 and 3909 strains. The inactivation of AdeB and AdeJ RND EPs in *A. baumannii* ATCC19606 increased the susceptibility to CHX and BZK alone or in the presence of 32 mg/L RV. Concordantly, carbonyl cyanide m-chlorophenylhydrazine (CCCP) increased the susceptibility to CHX, BZK and RV and dose-dependently inhibited biofilm formation in *A. baumannii* ATCC 19606, 4190 and 3909 strains. RV at 32 mg/L inhibited basal and CHX-induced EP genes expression, while increased EP gene expression in the presence of BZK during *A. baumannii* ATCC19606 biofilm growth. In addition, CHX and BZK alone or in combination dose-dependently reduced preformed biofilm of all *A. baumannii* strains. The combination of RV with CHX and BZK additively decreased minimal biofilm eradicating concentrations in *A. baumannii* strains.

**Conclusion:**

These results demonstrate that: (i) CHX and BZK alone or in the presence of RV inhibit biofilm growth and preformed biofilm in *A. baumannii*; (ii) tolerance to CHX and BZK during biofilm growth is dependent on the activation of AdeB and AdeJ EPs; and (iii) the inhibitory effect of RV on biofilm growth is mediated by the inhibition of EP genes expression in *A. baumannii*.

## 1 Introduction

*Acinetobacter baumannii* has been recognized as a common cause of severe infections and epidemics among hospitalized patients (Wong et al., 2017; Gaiarsa et al., 2019). The infections caused by *A. baumannii* are difficult to treat because the majority of *A. baumannii* isolates are multi-drug resistant (MDR) and a fraction of them are extensively-drug resistant (XDR), showing resistance to carbapenems and to tigecycline and/or colistin (Wong et al., 2017; Dickstein et al., 2019). Moreover, carbapenem-resistant *A. baumannii* has been included in the critical priority category among bacterial pathogens of public health importance by world health organization (WHO Bacterial Priority Pathogens List, 2024, 2024).

*A. baumannii* infections and spread in the contaminated environment are related to virulence features of the bacteria such as biofilm formation on infected host mucosa and contaminated abiotic surfaces (Lucidi et al., 2024). The ability to form biofilm is a common virulence-related trait of epidemic *A. baumannii* strains (Giannouli et al., 2013), which favors medical device-associated infections and survival in the contaminated environment (Lucidi et al., 2024).

The persistence of *A. baumannii* in the hospital environment is contributed also by tolerance of the bacteria to several biocides, which are used as antiseptics or disinfectants (McDonnell and Russell, 1999; Geraldes et al., 2023). Among them, the biguanide chlorhexidine (CHX) is a cationic compound, which acts by binding to the cell membrane through electrostatic interactions and inhibiting membrane enzymes (Geraldes et al., 2023). CHX is widely used for hand hygiene, skin antisepsis and oral care (Milstone et al., 2008; Geraldes et al., 2023). The quaternary ammonium compound benzalkonium chloride (BZK) is a cationic amphiphilic surface-active agent, which binds to the cellular membrane and disrupt the interaction between lipids and proteins, causing increased membrane permeability and leakage of intracellular components (Geraldes et al., 2023). BZK is frequently used for hospital disinfection and as an antiseptic in antimicrobial soaps (Merchel Piovesan Pereira and Tagkopoulos, 2019). *A. baumannii* strains are able to grow and retain viability in the presence of sub-inhibitory concentrations of CHX and of other biocides and are considered tolerant to them (Wisplinghoff et al., 2007; Rajamohan et al., 2010a). Tolerance to CHX and BZK has been increasingly reported in *A. baumannii* (Fernández-Cuenca et al., 2015; Li et al., 2023). Previous studies demonstrated that biocide tolerance in *A. baumannii* is dependent on the activation of efflux pumps (EPs) belonging to proteobacterial antimicrobial compound (PACE), resistance–nodulation–cell division (RND) and major facilitator superfamily (MFS) efflux systems (Rajamohan et al., 2010a,b; Hassan et al., 2013; Tucker et al., 2014; Yoon et al., 2015; Foong et al., 2019; Migliaccio et al., 2022a; Meyer et al., 2022; Li et al., 2023). The transcriptomic analysis of *A. baumannii* ATCC 17978 in response to CHX exposure identified *adeABC* and *aceI* as the most highly up-regulated genes under CHX stress and the product of *aceI* gene acting as an efflux system for CHX (Hassan et al., 2013). Also, the tolerance to CHX and other biocides in *A. baumannii* has been demonstrated to be contributed by the activation of EPs belonging to RND and PACE efflux systems (Rajamohan et al., 2010a; Tucker et al., 2014) and to MFS efflux system (Rajamohan et al., 2010b; Foong et al., 2019). In agreement with the above findings, Migliaccio et al. demonstrated that CHX and BZK tolerance in *A. baumannii* ATCC 19606 was mediated by the activation of AmvA MFS, AceI PACE, AdeJ and AdeB RND EPs systems, with AdeB playing a major role (Migliaccio et al., 2022a). Moreover, the authors found that the natural monomeric stilbenoid resveratrol (RV) (Mattio et al., 2020), at non-toxic concentrations, inhibited EPs gene expression and restored susceptibility to CHX and BZK biocides in *A. baumannii* ATCC19606 and in *A. baumannii* strains assigned to distinct genotypes (Migliaccio et al., 2022a,b).

Previous studies demonstrated that the susceptibility to biocides decreases in microbial biofilms respect to planktonic cells and that microbial biofilms are tolerant to biocides (Charron et al., 2023). Microbial biofilms on dry surfaces and medical devices are difficult to eradicate because of the reduced susceptibility to biocides and represent a major problem of infection control in healthcare settings (Maillard and Centeleghe, 2023). Mounting evidence indicate that biofilm growth contributes to the fitness of *A. baumannii* in the infected host and in the contaminated hospital environment (Lucidi et al., 2024). Also, a recent study demonstrated that sub-inhibitory concentrations of the biocides can promote *A. baumannii* tolerance to antibiotics that act intracellularly and suggested a positive association between biocide tolerance and antimicrobial resistance in *A. baumannii* (Li et al., 2023). The susceptibility to biocides of *A. baumannii* biofilms and the mechanisms responsible for the tolerance to biocides of *A. baumannii* biofilms have not yet been investigated in detail.

The objectives of the present study were to: (i) analyze the susceptibility to CHX and BZK biocides in *A. baumannii* during biofilm growth and on preformed biofilms; (ii) assess whether RV may revert tolerance and restore susceptibility to CHX and BZK of *A. baumannii* biofilms; and (iii) evaluate the contribution of EPs to these phenomena.

## 2 Materials and methods

### 2.1 Bacterial strains and culture conditions

The bacterial strains used in this work were listed in Supplementary Table S1. All strains were cultured under aerobic conditions at 37°C in Luria-Bertani (LB) broth and agar. Cation-adjusted Mueller-Hinton broth (CAMHB) and Tryptic soy broth (TSB) were used to perform susceptibility tests and biofilm assays. The chemical reagents were chlorhexidine digluconate (CHX), the quaternary ammonium compounds benzalkonium chloride (alkylbenzyldimethylammonium chloride, BZK), carbonyl cyanide m-chlorophenylhydrazine (CCCP), and resveratrol (3,5,4′-trihydroxy-trans-stilbene, RV). The antimicrobials and chemical reagents were purchased from Sigma-Aldrich (Sigma, Milan, Italy). Stock solution of RV was prepared in dimethyl sulfoxide (DMSO) at a concentration of 65 mg/mL, while the CHX and BZK were dissolved in H_2_O at a concentration of 200 and 100 mg/mL, respectively.

### 2.2 Determination of minimum inhibitory concentration (MIC)

The MIC values of CHX, BZK, and RV were determined by a manual microdilution method, according to the recommended procedures by the European Committee for Antimicrobial Susceptibility Testing (EUCAST) (Giske et al., 2020). Briefly, bacterial suspensions were adjusted to 0.5 McFarland using a BD PhoenixSpec nephelometer after an overnight growth in LB. Subsequently, the bacterial cells were diluted in CAMHB and placed into a polystyrene 96-well plate to a final culture density of approximately 1 × 10^5^ CFU. Two-fold serial dilutions of CHX (1–256 mg/L), BZK (1–256 mg/L) and RV (32–1,024 mg/L) were prepared and added to wells containing bacterial cells. Wells containing CAMHB only were used as negative control wells, and wells containing bacterial cells without additional compounds were used as positive controls for growth. The microplates were incubated at 37°C for 18–24 h. The MIC was determined as the minimum compound concentration needed to inhibit visible bacterial growth. The strains showing CHX and BZK MIC values <4 mg/L were considered susceptible, while the strains having MIC values ≥4 mg/L were considered tolerant (Rajamohan et al., 2010a). All tests were performed in triplicate and repeated three times.

### 2.3 Biofilm assays

Biofilm formation and minimal biofilm inhibitory concentration (MBIC) were assessed using a crystal violet (CV) staining assay as previously described (Vollaro et al., 2020; Macià et al., 2014) and calculated according to the European Committee for Antimicrobial Susceptibility Testing (EUCAST) (Giske et al., 2020). Bacterial cell suspensions were adjusted to 0.5 McFarland using a BD PhoenixSpec nephelometer after an overnight growth in TSB. Subsequently, the bacterial cells were diluted to a final culture density of approximately 1 × 10^6^ CFU/mL in TSB and were transferred into a 96-well flat-bottomed polystyrene microtiter plate containing scalar doses of CHX and BZK (1–256 mg/L) alone or in presence of RV (32 and 64 mg/L) or CCCP (0.5, 1 and 2 mg/L). Untreated bacteria were incubated with 100 μL of broth and used as a control. The microplates were incubated at 37°C for 24 h. The culture supernatants were gently discarded, the wells were washed twice with phosphate buffered saline (PBS, Sigma, Milan, Italy) pH 7.4 and the biofilms were stained with 200 μL of 0.1% CV for 20 min. The wells were washed twice with PBS, and dye was re-eluted with ethanol. The absorbance was measured at 595 nm using a microplate reader (Bio-Rad Laboratories S.r.l.). The OD_595_/OD_600_ ratio was used to normalize the amount of biofilm formed versus O/N growth. The MBIC was determined as the minimum compound concentration required to inhibit sessile growth. *p*-values were calculated using two-way ANOVA and Tukey tests (**p < 0.01 and ***p < 0.001) on the GraphPad Prism software.

Air-liquid interface biofilm formation (ALIB) was examined as described by Nait Chabane et al. (2014). Briefly, bacterial cell suspension was prepared at 1 McFarland and diluted 1:100 in sterile polystyrene tubes (13×75 mm) containing 2 mL of TSB with RV in presence or absence of the ½ MIC of CHX and/or BZK. Cultures were incubated for 72 h at 37°C without shaking. After rinsing with water, tubes were stained by incubation with 0.1% crystal violet for 20 min. Crystal violet was washed twice with PBS pH 7.4 and solubilized with ethanol. The OD595/OD600 ratio was used to normalize the amount of air-liquid biofilm formed. Assays were performed in triplicate. *p* values were obtained by two-way ANOVA and Bonferroni correction (*p < 0.05; **p < 0.01; ***p < 0.001) using the GraphPad Prism software.

Preformed biofilm biomasses were grown for 24 h as described above. After treatments, CV staining was performed to assess the biofilm biomass as previously described (Vollaro et al., 2020). The minimum biofilm eradication concentration (MBEC) of preformed biofilm was investigated as described by Macià et al. (2014) and calculated according to the European Committee for Antimicrobial Susceptibility Testing (EUCAST) (Giske et al., 2020). The cell viability of preformed biofilms was assessed through the analysis of the metabolic activity as previously described (Ahmed et al., 2016). Briefly, the preformed biofilms were grown in microplates as described above, washed with PBS to remove planktonic cells, and then scalar doses of CHX and BZK alone or in presence of 32 and 64 mg/L RV were added. The 96-well microtiter plate was re-incubated at 37°C for 24 h. Thereafter, the supernatant was gently discarded, the wells were washed twice with PBS and MTT reagent (3–2,5-diphenyltetrazolium bromide) was added to each well. Therefore, the microtiter plate was incubated at 37°C for 3 h in the dark to promote the reduction of MTT. The OD_490_/OD_600_ ratio was used to normalize the amount of preformed biofilm. The assays were performed in triplicate. The MBEC was calculated as the lowest concentration of a compound required to completely eradicate preformed biofilm. *p* values were obtained by two way ANOVA and adjusted by the Bonferroni correction (*p < 0.05; **p < 0.01; ***p < 0.001) using GraphPad Prism version 8.0 for Windows (GraphPad Software, San Diego, CA, United States).

Synergy between biocides and RV to inhibit or eradicate biofilm was determined using a checkerboard titration assay as previously described (Hall et al., 1983). The combined effects were then determined by calculating the fractional inhibitory concentration index (FICI) as follows: FICI = FIC_A_ + FIC_B_, where FIC_A_ is the ratio of the MBIC (or MBEC) of CHX and BZK with RV (0 or 32 or 64 mg/L) in combination and the MBIC (or MBEC) of CHX with RV (0 or 32 or 64 mg/L) alone, and FIC_B_ is the ratio of the MBIC (or MBEC) CHX and BZK with RV (0 or 32 or 64 mg/L) combination and the MBIC (or MBEC) of BZK with RV (0 or 32 or 64 mg/L) alone (*Σ* FIC = [(MBIC (or MBEC) CHX + BZK) + 0–32-64 RV/ MBIC (or MBEC) CHX] + [(MBIC (or MBEC) BZK + CHX) + 0–32-64 RV / MBIC (or MBEC) BZK]). The FIC index was interpreted as synergy (FICI ≤0.5), additivity (FICI >0.5 to ≤1.0), indifference (FICI >1.0 to ≤2.0) and antagonism (FICI >2.0) (Odds, 2003).

### 2.4 RNA purification and real-time RT-PCR

*Acinetobacter baumannii* ATCC 19606 was prepared by growing in TSB overnight and adjusting the turbidity to 0.5 McFarland. The bacterial cells were then diluted to a final culture density of approximately 1 × 10^6^ CFU/mL in TSB and were transferred into a 12-well flat-bottomed polystyrene microtiter plate containing CHX or BZK subMICs in the presence or absence of 32 mg/L RV. The bacteria incubated with TSB were used as untreated controls. The plates were incubated at 37°C for 24 h. The plates were then washed with PBS to remove planktonic cells and the adherent cells were scraped with a pipettor and total RNA was isolated via TRIzol reagent (Qiagen, Milan, Italy). The cDNAs were synthesized using QuantiTect Reverse Transcription Kit (Qiagen, Milan, Italy), according to the manufacturer’s protocol. Real-time RT-PCR assays were performed using SYBR Green master mix (Applied Biosystems) (Esposito et al., 2019). The *rpoB* housekeeping gene was used to normalize the expression of target genes as previously shown (Armalytė et al., 2023). The fold-changes of gene expression levels were calculated using the 2–ΔΔct method (Livak and Schmittgen, 2001). All experiments were performed three times in triplicate.

### 2.5 Cytotoxicity assay

The cytotoxicity of CHX, BZK and RV was examined as described by Ebbensgaard et al. (2018). Briefly, fresh defibrinated horse blood (Oxoid, Milan, Italy) was washed three times with PBS, centrifuged for 5 min at 500 rpm and suspended in PBS pH 7.4 to remove the plasma and mononuclear cells. The washed erythrocytes were transferred to a 96 well microtiter plate and incubated in the presence of 32 or 64 mg/L RV, 32 mg/L CHX and 32 mg/L BZK corresponding to MBICs, 512 mg/L CHX and 64 mg/L BZK corresponding to CHX MBEC and BZK MBEC, respectively. PBS was used as a negative control, and 1% (v/v) TritonX100 was used as a positive control. The microtiter plates were incubated for 60 min at 37°C and the supernatants were transferred to a 96 well polystyrene microtiter plate. The hemoglobin release was monitored by measuring the absorbance at 540 nm. The assays were performed in triplicate and repeated twice.

### 2.6 Statistical analysis

Statistical analyses were carried out using GraphPad Prism version 8.0 for Windows (GraphPad Software, San Diego, CA, United States). Pearson correlation coefficient was used to analyze the correlation between EPs gene expression level and biofilm formation ability of the EPs isogenic mutants. All experiments were performed at least three times and the results were shown as means +/− SD. Differences between mean values were tested for significance using ANOVA adjusted by Tuckey test (*p < 0.05; **p < 0.01; ***p < 0.001).

## 3 Results

### 3.1 Effect of CHX, BZK and RV combinations on biofilm formation in *Acinetobacter baumannii*

The effects of CHX, BZK and CHX + BZK alone or in combination with RV on *A. baumannii* biofilm formation was investigated in *A. baumannii* ATCC 19606 reference strain (Janssen et al., 1997) and in *A. baumannii* 4190 and 3909 carbapenem resistant and multi-drug resistant epidemic strains (Zarrilli et al., 2011) (Supplementary Table S1). *A. baumannii* ATCC 19606, 4190 and 3909 strains belonged to distinct genotypes (Di Popolo et al., 2011) and all showed high biofilm forming capability (Giannouli et al., 2013). In all three *A. baumannii* strains, BZK at concentrations of ¼ and ½ MIC significantly (*p < 0.05 and **p < 0.01, respectively) inhibited biofilm formation by 35 and 45%, respectively. Also, CHX at ¼ and ½ MIC significantly (**p < 0.01 and ***p < 0.001, respectively) inhibited *A. baumannii* 4190 biofilm growth by 50 and 60%, respectively. Furthermore, the combination of CHX + BZK at all sub-MIC concentrations dose-dependently and significantly (*p < 0.05, **p < 0.01 and ***p < 0.001) inhibited biofilm formation in all three *A. baumannii* strains (Table 1). To investigate whether RV had a combinatorial effect with CHX or BZK, the bacterial suspensions were cultured in the presence of CHX or BZK subMICs and RV at 32 or 64 mg/L concentrations. The addition of RV to CHX or BZK increased the inhibition of *A. baumannii* biofilm growth by 10–40%, while RV at 32 and 64 mg/L alone did not inhibit significantly (*p* > 0.05) biofilm formation. Interestingly, the inhibitory effect on biofilm formation of the CHX + BZK combination increased in the presence of RV dose-dependently and significantly (*p < 0.05, **p < 0.01, and ***p < 0.001) in all three strains. Moreover, the combinations of ¼ MIC CHX + BZK in the presence of 32 mg/L RV and 1/8 MIC CHX + BZK in the presence of 64 mg/L RV were able to stop completely and significantly (*p* < 0.001) biofilm growth (Table 1). All the concentrations of CHX, BZK and RV used were not cytotoxic (Supplementary Figure S1). These results demonstrated the synergistic effect of the combination of CHX + BZX and RV (FICI <0.5) on the inhibition of biofilm formation in *A. baumannii* ATCC19606, 4190 and 3909 strains.

**Table 1:** Effect of 32 and 64 mg/L RV in presence or absence of CHX and BZK sub-MICs concentrations, alone or in combination, on biofilm formation in *A. baumannii* ATCC 19606, 4190 and 3909.

		Strains		
	Treatment	ATCC 19606	4190	3909
	TSB	1.02 ± 0.05	0.98 ± 0.02	1 ± 0.02
	32 RV	0.74 ± 0.06	0.89 ± 0.04	0.86 ± 0.02
	64 RV	0.73 ± 0.09	0.84 ± 0.06	0.84 ± 0.1
0 RV	1/8 CHX MIC	0.99 ± 0.01	0.87 ± 0.06	0.9 ± 0.1
	1/4 CHX MIC	0.98 ± 0.03	0.49 ± 0.03**	0.79 ± 0.03
	1/2 CHX MIC	0.87 ± 0.08	0.4 ± 0.1***	0.81 ± 0.03
	1/8 BZK MIC	0.74 ± 0.01	0.69 ± 0.06*	0.8 ± 0.1
	1/4 BZK MIC	0.64 ± 0.1*	0.7 ± 0.04*	0.64 ± 0.03*
	1/2 BZK MIC	0.67 ± 0.04*	0.54 ± 0.03**	0.57 ± 0.03**
	1/8 CHX MIC+1/8 BZK MIC	0.73 ± 0.01	0.57 ± 0.06*	0.46 ± 0.02***
	1/4 CHX MIC+1/4 BZK MIC	0.5 ± 0.03**	0.57 ± 0.03*	0.2 ± 0.01***
	½ CHX MIC+ ½ BZK MIC	0.41 ± 0.03***	0.26 ± 0.02***	0.1 ± 0.03***
32 RV	1/8 CHX MIC	0.75 ± 0.01	0.49 ± 0.06***	0.85 ± 0.06
	1/4 CHX MIC	0.70 ± 0.04	0.44 ± 0.01***	0.77 ± 0.04
	1/2 CHX MIC	0.73 ± 0.04	0.4 ± 0.05***	0.64 ± 0.03
	1/8 BZK MIC	0.71 ± 0.02	0.59 ± 0.06*	0.6 ± 0.03*
	1/4 BZK MIC	0.64 ± 0.03*	0.57 ± 0.03*	0.64 ± 0.02*
	1/2 BZK MIC	0.67 ± 0.02*	0.54 ± 0.01**	0.57 ± 0.03**
	1/8 CHX MIC+1/8 BZK MIC	0.44 ± 0.02***	0.35 ± 0.06***	0.46 ± 0.03***
	1/4 CHX MIC+1/4 BZK MIC	0 ± 0.02***	0 ± 0.02***	0 ± 0.01***
	½ CHX MIC+ ½ BZK MIC	0 ± 0.02***	0 ± 0.01***	0 ± 0.02***
64 RV	1/8 CHX MIC	0.69 ± 0.02*	0.47 ± 0.03***	0.74 ± 0.03
	1/4 CHX MIC	0.56 ± 0.07*	0.4 ± 0.08***	0.65 ± 0.06*
	1/2 CHX MIC	0.46 ± 0.02***	0.39 ± 0.02***	0.52 ± 0.03**
	1/8 BZK MIC	0.59 ± 0.02*	0.56 ± 0.03**	0.6 ± 0.03**
	1/4 BZK MIC	0.58 ± 0.01*	0.54 ± 0.07**	0.57 ± 0.02**
	1/2 BZK MIC	0.57 ± 0.03*	0.44 ± 0.04**	0.55 ± 0.02**
	1/8 CHX MIC+1/8 BZK MIC	0 ± 0.02***	0 ± 0.02***	0 ± 0.02***
	1/4 CHX MIC+1/4 BZK MIC	0 ± 0.01***	0 ± 0.04***	0 ± 0.1***
	½ CHX MIC+ ½ BZK MIC	0 ± 0.02***	0 ± 0.03***	0 ± 0.02***

The results are shown as OD_595_/OD_600_; Synergistic inhibitory effects of RV and/or CHX and BZK on biofilm formation are shown in bold; *p*-values were calculated using two-way ANOVA (*p < 0.05; **p < 0.01; ***p < 0.001). Assays were performed in triplicate.

### 3.2 Resveratrol effect in presence of CHX and BZK on biofilm formation at air-liquid interface (ALIB)

**Figure 1:**
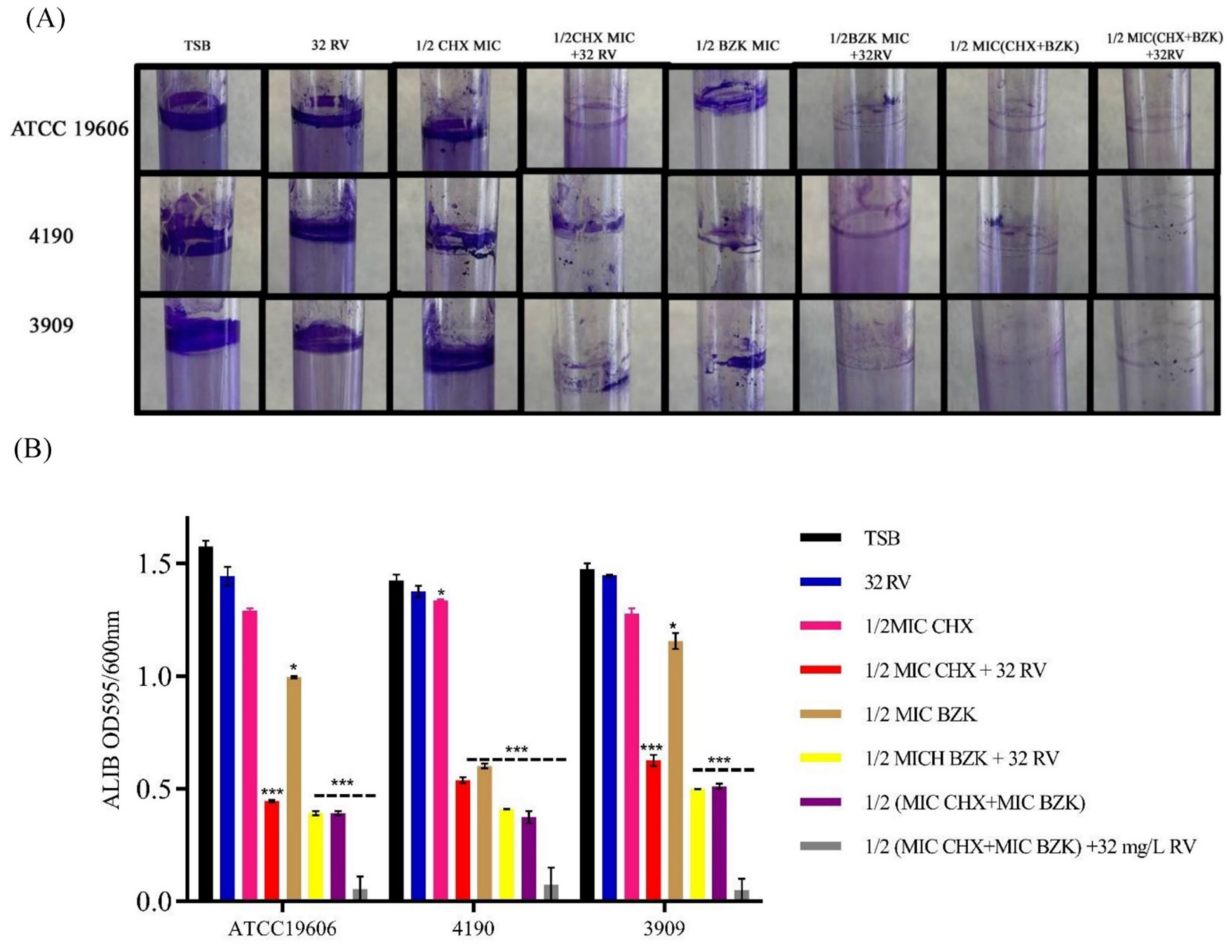
Effect of resveratrol alone or in combination with CHX and BZK on ALIB in *A. baumannii* ATCC19606, 4190 and 3909. **(A)** *A. baumannii* ATCC19606 biofilm was exposed to RV in the presence or absence of ½ MIC CHX (16 mg/L) and/or ½ MIC BZK (8 mg/L). *A. baumannii* 4190 and 3909 biofilms were exposed to RV in presence or absence of ½ CHX and/or BZK MICs corresponding to16 and 4 mg/L, respectively. **(B)** CV staining assay of ALIB in the presence of 0 and 32 mg/L of RV with CHX and BZK sub-MICs. *p*-values were calculated using ANOVA (*p < 0.05, **p < 0.01, and ***p < 0.001). Assays were performed in triplicate.

*Acinetobacter baumannii* ATCC 19606, 4190 and 3909 strains had the ability to form a thin pellicle and a ring biofilm at air-liquid interface by adhering to the surface of the liquid medium (Nait Chabane et al., 2014; Figure 1A). Thus, the effect of CHX, BZK and RV alone or in combination on ring biofilm formation of *A. baumannii* strains was evaluated. The treatment with CHX at ½ MIC or RV did not inhibit ring biofilm formation in all three strains tested. Instead, ½ BZK MIC significantly reduced ALIB compared with untreated controls by 40, 70 and 20% in *A. baumannii* ATCC19606, 4190 and 3909 strains, respectively. Interestingly, a reduction of 70–80% in ALIB was observed in all three strains following treatment with ½ CHX or BZK in the presence of 32 mg/L RV. Moreover, the maximum inhibitory effect (95%) on the formation of biofilm rings was shown by the combination of CHX and BZK at sub-MIC concentrations in the presence of 32 mg/L RV (Figure 1B).

### 3.3 Role of efflux pumps on *Acinetobacter baumannii* ATCC 19606 biofilm formation in the presence of CHX, BZK and RV

Because it has been demonstrated that tolerance to CHX and BZK during planktonic growth of *A. baumannii* is mediated by the activation of AceI, AdeB and AmvA EPs (Migliaccio et al., 2022a), we analyzed the effect of EPs inactivation on the susceptibility of ATCC 19606 biofilm formation to CHX, BZK and RV (Table 2). As shown in Table 2, biofilm formation of *A. baumannii* ATCC 19606 isogenic mutants *ΔadeB* and *ΔadeJ*, belonging to Resistance-Nodulation-Division (RND) superfamily, *ΔaceI* belonging to Proteobacterial antimicrobial compound efflux (PACE) superfamily and *ΔamvA* belonging to major facilitator (MFS) superfamily, was reduced by 41, 46, 24, and 43%, respectively, compared to parental cells. Also, 1/8, ¼ and ½ CHX MICs alone or in the presence of 32 mg/L RV did not reduce biofilm growth in single EPs mutants (Table 2). On the other hand, sub-MIC doses of BZK (¼ and ½ BZK MIC) significantly reduced biofilm growth in ATCC 19606 wild type and *ΔadeB* or *ΔadeJ* ATCC 19606 mutants. Furthermore, the combination of CHX and BZK at 1/8 MICs completely inhibited biofilm formation in *ΔadeJ*. Also, CHX and BZK combination at ¼ MICs was able to completely inhibit biofilm formation in *ΔadeB* and *ΔadeJ* mutants, while ½ MICs CHX and BZK combination inhibited biofilm growth in all EPs mutants of ATCC 19606. Interestingly, the addition of 32 mg/L RV to 1/4 sub-MICs CHX and BZK combination inhibited biofilm formation in ATCC19606 and in all EPs mutants (Table 2).

**Table 2:** Effect of 32 and 64 mg/L RV in presence or absence of CHX and BZK sub-MIC concentrations, alone or in combination, on biofilm formation in *A. baumannii* ATCC 19606 and isogenic mutants of AmvA, AdeB, AdeJ, AceI EPs.

		Strains				
Treatment		ATCC 19606	*∆adeB*	*∆adeJ*	*∆aceI*	*∆amvA*
	TSB	1.02 ± 0.05	0.59 ± 0.03	0.54 ± 0.01	0.76 ± 0.06	0.57 ± 0.06
	32 RV	0.74 ± 0.06	0.54 ± 0.06	0.63 ± 0.03	0.78 ± 0.01	0.69 ± 0.06
0 RV	1/8 CHX MIC	0.99 ± 0.01	0.52 ± 0.01	0.61 ± 0.03	0.71 ± 0.03	0.50 ± 0.03
	1/4 CHX MIC	0.98 ± 0.03	0.49 ± 0.01	0.55 ± 0.03	0.61 ± 0.04	0.50 ± 0.01
	1/2 CHX MIC	0.87 ± 0.08	0.47 ± 0.02	0.53 ± 0.03	0.54 ± 0.03	0.53 ± 0.04
	1/8 BZK MIC	0.74 ± 0.01	0.54 ± 0.06	0.54 ± 0.03	0.77 ± 0.04	0.34 ± 0.04
	1/4 BZK MIC	0.64 ± 0.1*	0 ± 0.02***	0.21 ± 0.03**	0.59 ± 0.04	0.34 ± 0.01
	1/2 BZK MIC	0.67 ± 0.04*	0 ± 0.02***	0 ± 0.02***	0.61 ± 0.04	0.35 ± 0.01
	1/8 CHX MIC+1/8 BZK MIC	0.73 ± 0.01	0.53 ± 0.01	0 ± 0.1***	0.64 ± 0.03	0.45 ± 0.01
	1/4 CHX MIC+1/4 BZK MIC	0.5 ± 0.03**	0 ± 0.02***	0 ± 0.03***	0.19 ± 0.06***	**0.42 ± 0.06**
	½ CHX MIC+ ½ BZK MIC	0.41 ± 0.03**	0 ± 0.1***	0 ± 0.1***	0 ± 0.03***	0 ± 0.03***
32 RV	1/8 CHX MIC	0.75 ± 0.01	0.5 ± 0.02	0.60 ± 0.04	0.42 ± 0.03*	0.52 ± 0.04
	1/4 CHX MIC	0.70 ± 0.04	0.49 ± 0.02	0.51 ± 0.04	0.45 ± 0.03*	0.50 ± 0.01
	1/2 CHX MIC	0.73 ± 0.04	0 ± 0.04***	0.42 ± 0.01	0.41 ± 0.01*	0.42 ± 0.01
	1/8 BZK MIC	0.71 ± 0.0*	0.55 ± 0.02	0.59 ± 0.06	0.70 ± 0.04	0.45 ± 0.01
	1/4 BZK MIC	0.64 ± 0.03*	0 ± 0.02***	0 ± 0.02***	0.62 ± 0.01	0.44 ± 0.03
	1/2 BZK MIC	0.67 ± 0.02*	0 ± 0.1***	0 ± 0.01***	0.40 ± 0.04*	0.43 ± 0.01
	1/8 CHX MIC+1/8 BZK MIC	0.44 ± 0.02**	0 ± 0.02***	0 ± 0.04***	**0.52 ± 0.03**	**0.54 ± 0.04**
	1/4 CHX MIC+1/4 BZK MIC	0 ± 0.02***	0 ± 0.1***	0 ± 0.01***	0.0 ± 0.01***	0.0 ± 0.03***
	1/2 CHX MIC+1/2 BZK MIC	0 ± 0.02***	0 ± 0.03***	0 ± 0.03***	0 ± 0.03***	0 ± 0.03***

The results are shown as OD595 nm/600 nm; Synergistic inhibitory effects of RV and/or CHX and BZK on biofilm formation are shown in bold; *p*-values were calculated using two-way ANOVA (*p < 0.05; **p < 0.01; ***p < 0.001). Assays were performed in triplicate.

**Figure 2:**
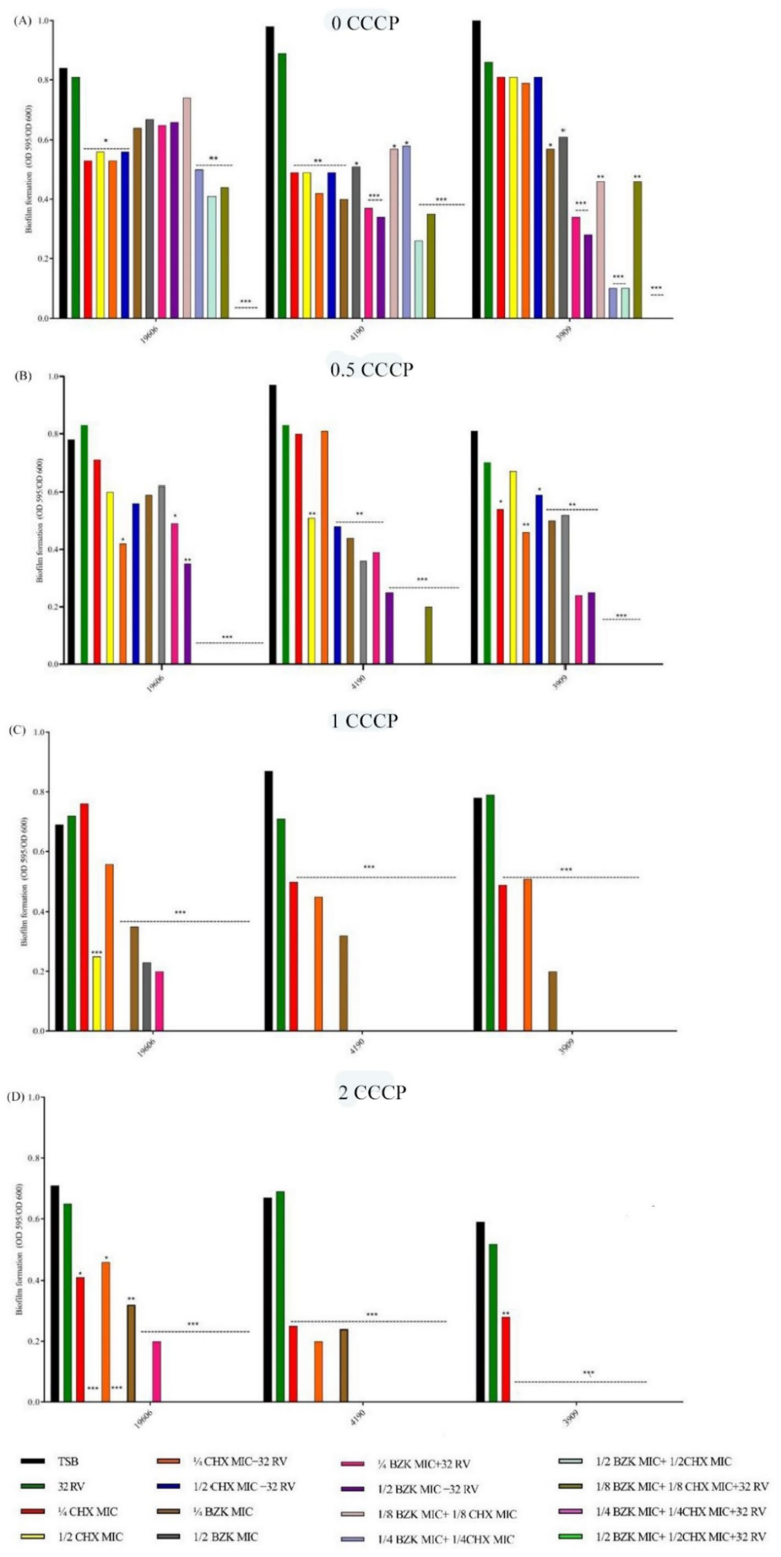
Effect of CCCP at 0, 0.5, 1, and 2 mg/L and CHX, BZK and RV combinations on biofilm formation in *A. baumannii*. ATCC 19606 biofilm growth was performed in the presence or absence of CCCP, and RV and/or ½, ¼ and 1/8 MIC CHX (16, 8, and 4 mg/L) and/or ½, ¼, and 1/8 MIC BZK (8, 4, and 2 mg/L). *A. baumannii* 4190 and 3909 biofilms were exposed to CCCP and RV in the presence or absence of ½, ¼, and 1/8 MIC CHX (16, 8, and 4 mg/L) and/or ½, ¼, and 1/8 MIC BZK (4, 2, and 1 mg/L). *p*-values were calculated using ANOVA (*p < 0.05, **p < 0.01, and ***p < 0.001). Assays were performed in triplicate.

The effect of EPs inhibition was then investigated in *A. baumannii* strains treated without and with the synthetic inhibitor of proton motive-force-dependent pumps CCCP at non-toxic concentrations of 0.5, 1 and 2 mg/L. As shown in Figure 2, CCCP dose-dependently inhibited biofilm formation in the presence of CHX and BZK biocides alone or in combinations with RV in all strains. In particular, CCCP at 0.5 mg/L completely inhibited biofilm growth following treatment with either CHX at ½ MIC or BZK at ½ MIC in the presence of 32 mg/L RV in ATCC19606 and 3909, while CCCP at 0.5 mg/L completely inhibited biofilm growth in the presence of the combination of CHX at ½ MIC, BZK at ½ MIC and 32 mg/L RV. Whereas, CCCP at 1 mg/L completely inhibited biofilm growth following treatments with either CHX alone at ½ MIC in all three strains, BZK alone at ½ MIC in 4190 and 3909 strains or BZK alone at ¼ MIC in ATCC 19606 strain. Moreover, CCCP at 2 mg/L totally inhibited biofilm formation following treatments with ¼ CHX or BZK MIC used as single agents in all three strains (Figure 2). The above data indicate that the susceptibility to biocides in *A. baumannii* biofilm growth is contributed by the activity of EPs.

### 3.4 Effect of RV on *amvA, adeB, adeJ, aceI* gene expression during biofilm formation in the presence or absence of CHX and BZK sub-MIC concentrations in *Acinetobacter baumannii* ATCC 19606

**Figure 3:**
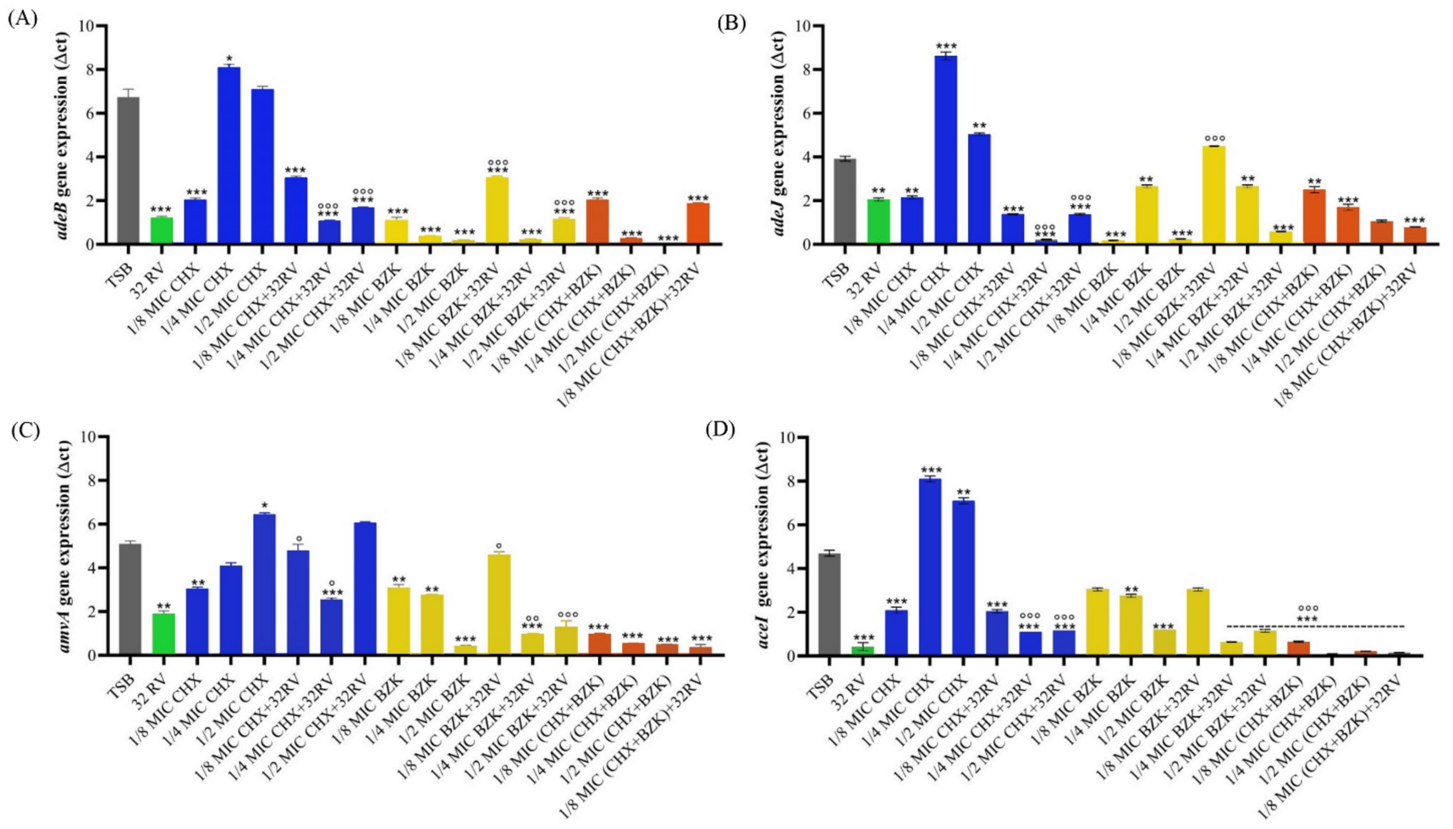
Effect of resveratrol and CHX or BZK on EPs gene expression during biofilm formation in *A. baumannii* ATCC19606. Effect of 32 mg/L RV in presence or absence of ½, ¼, and 1/8 MIC CHX (16, 8, and 4 mg/L) and/or ½, ¼, and 1/8 MIC BZK (8, 4, and 2 mg/L) on *adeB* **(A)**, *adeJ* **(B)**, *amvA* **(C)** and *aceI* **(D)** genes expression. *p*-values were calculated using ANOVA (*p < 0.05, **p < 0.01 and ***p < 0.001 vs. TSB and °*p* < 0.05, °°*p* < 0.01 and °°°*p* < 0.001 vs. CHX or BZK alone). Assay was performed in triplicate.

We previously demonstrated that the tolerance to CHX and BZK biocides during *A. baumannii* planktonic growth was stimulated by the expression of EPs and that RV at non-toxic concentrations negatively regulated it (Migliaccio et al., 2022a,b). Herein, we analyzed the expression of EPs genes during biofilm growth of *A. baumannii* and assessed whether RV and biocides treatments are able to modulate it. The *amvA*, *adeB*, *adeJ*, *aceI* genes were upregulated by 26-, 10-, 10.5- and 6.7-folds, respectively, during biofilm growth compared with planktonic cell growth (Supplementary Figure S2). Also, the expression of all EPs genes was inhibited by 32 mg/L RV and 1/8 CHX MIC during biofilm growth. In contrast, ¼ and ½ CHX MICs induced the expression of *adeB, adeJ* and *aceI*, ½ CHX MIC induced *amvA* expression. Interestingly, CHX sub-MICs concentrations in the presence of 32 mg/L RV inhibited both basal and CHX-induced expression of *adeB*, *adeJ* and *aceI* EPs genes (Figure 3). Also, BZK sub-MICs dose-dependently and significantly inhibited *amvA*, *adeB*, *adeJ*, *aceI* gene expression, with the exception of *adeJ* gene expression in the presence of ¼ BZK MIC. Interestingly, the combination of 32 mg/mL RV and BZK at 1/8 subMIC increased *adeB, adeJ and amvA* expression compared with BZK alone. Moreover, the combination of CHX and BZK, in the presence or absence of RV, inhibited *adeB*, *adeJ*, *amvA* and *aceI* gene expression (Figure 3).

Also, the correlations among *amvA, adeB, adeJ, aceI* expression and biofilm formation in ATCC 19606 EPs isogenic mutants and parental cells treated with CHX and BZK in the presence or absence of RV was analyzed using Pearson correlation test. A positive correlation was found between biofilm formation and *adeB* EP gene expression (*r* = 0.4), while the expression of *adeJ*, *amvA* and *aceI* genes showed no correlation with biofilm formation (*r* = 0.1, *r* = 0.3, *r* = 0.1, respectively) (Supplementary Figure S3).

### 3.5 Effect of CHX, BZK and RV combinations on *Acinetobacter baumannii* preformed biofilms

**Figure 4:**
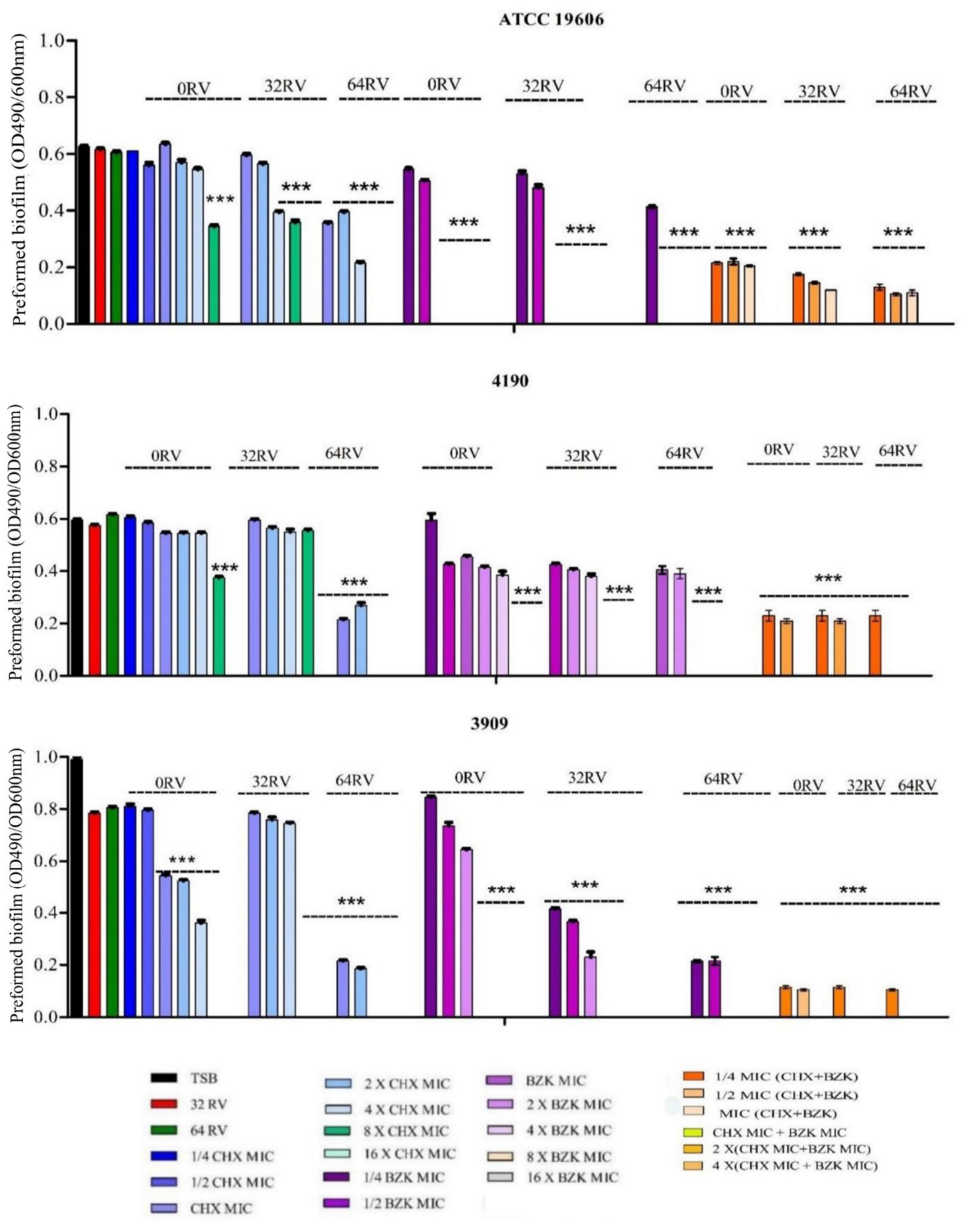
Effect of resveratrol in the presence of CHX and/or BZK on *A. baumannii* preformed biofilm. *A. baumannii* ATCC19606 preformed biofilm was exposed to 32 and 64 mg/L RV in the presence or absence of 1/4-16xMIC CHX (8–512 mg/L) and/or 1/4-16xMIC BZK (4–256 mg/L). *A. baumannii* 4190 and 3909 preformed biofilms were exposed to 32 and 64 mg/L RV in the presence or absence of 1/4-16xMIC CHX (8–512 mg/L) and/or 1/4-16xMIC BZK (2–128 mg/L). *p*-values were calculated using ANOVA (*p < 0.05, **p < 0.01, and ***p < 0.001). Assay was performed in triplicate.

The effects of RV, CHX and BZK on preformed biofilm of *A. baumannii* ATCC 19606, 3909 and 4190 strains were also analyzed (Table 3 and Figure 4). The results in Table 3 and Figure 4 showed that 32 and 64 mg/mL of RV and 8–128 mg/L of CHX, corresponding to ¼–4x CHX MICs, did not affect preformed biofilm in *A. baumannii* ATCC 19606 and 4190 strains, while 256 and 512 mg/L of CHX, corresponding to 8x and 16x CHX MICs, reduced preformed biofilm by 30 and 100%, respectively (Figure 4 and Table 3). In *A. baumannii* 3909, 32 and 64 mg/mL of RV and 8–16 mg/L of CHX, corresponding to ¼–½ CHX MICs, respectively, did not affect performed biofilm. On the other hand, 32, 64, 128 and 256 mg/L CHX, corresponding to 1x, 2x, 4x and 8x CHX MIC, respectively, decreased preformed biofilm by 40, 42, 60 and 100% (Figure 4). Interestingly, BZK showed higher effect on preformed biofilm than CHX, being able to inhibit dose-dependently preformed biofilm at 1x and 2x MICs and eradicate preformed biofilm at 4x MIC in *A. baumannii* ATCC 19606, 3909 and 4190 strains (Figure 4 and Table 3). Also, RV at 64 mg/L of, but not at 32 mg/L, decreased by 2-folds the MBEC of CHX and BZK in *A. baumannii* ATCC 19606, 4190 and 3909 strains (Table 3 and Figure 4). The combination of CHX and BZK reduced the MBEC values of all strains, with a greater effect on CHX MBEC (Table 3). In particular, the combination of CHX and BZK reduced CHX MBEC by 4-folds in 3909 and by 8-folds in ATCC 19606 and 4190, respectively, while reduced BZK MBEC by 2-folds in 4190 and did not affect BZK MBEC in ATCC 19606 and 3909 (Table 3). Moreover, RV at 32 and 64 mg/mL combined with CHX and BZK decreased MBEC values to 8 mg/L and 32 mg/L for BZK and CHX, respectively, in all strains analyzed (Table 3). The concentrations of CHX and BZK used to eradicate preformed biofilms were not cytotoxic (Supplementary Figure S1). The above all data demonstrated that the combination of CHX and BZK in the presence of RV had an additive effect on CHX and BZK MBEC and was able to eradicate preformed biofilms in *A. baumannii* strains (Figure 4 and Table 3).

**Table 3:** CHX and BZK MBEC values (mg/L) alone or in combination with 32 and 64 mg/L RV in *A. baumannii* ATCC 19606, 4190 and 3909.

	ATCC 19606			4190			3909		
	CHX	BZK	CHX + BZK	CHX	BZK	CHX + BZK	CHX	BZK	CHX + BZK
0 RV	512	16	64 + 16	512	64	64 + 16	256	16	64 + 16
32 RV	512	16	**32 + 8**	512	64	**32 + 8**	256	16	**32 + 8**
64 RV	256	8	**32 + 8**	128	32	**32 + 8**	128	8	**32 + 8**

Additive effects of eradication on preformed biofilms by CHX and BZK in the presence of RV are shown in bold. Assays were performed in triplicate.

## 4 Discussion

The ability to form sturdy biofilms (Lucidi et al., 2024) and the tolerance to biocides used as antiseptics or disinfectants (Fernández-Cuenca et al., 2015; Li et al., 2023) are specific features of *A. baumannii* that sustain its survival and spread in the contaminated hospital settings (Lucidi et al., 2024). The present study analyzes the effect of CHX and BZK biocides combinations in the presence of the natural monomeric stilbenoid RV on the inhibition of biofilm formation and eradication of preformed biofilms by three *A. baumannii* strains assigned to distinct genotypes, all showing elevated biofilm growth. Our data demonstrate that CHX and BZK biocides in contrast to their effects on planktonic growth (Migliaccio et al., 2022a) have different effects on biofilm growth. Sub-MIC doses of CHX alone had no effect on biofilm formation, while sub-MIC doses of BZK alone were able to decrease biofilm formation in *A. baumannii* strains. On the contrary, but in accordance with the effects found on planktonic growth (Migliaccio et al., 2022b), the combination of CHX and BZK at sub-MIC doses inhibited biofilm growth. Moreover, RV had a synergistic effect on their action in all three *A. baumannii* strains. Similarly, CHX, BZK and RV dose-dependently inhibited biofilm formation at air-liquid interface in all three *A. baumannii* strains.

There is increasing evidence demonstrating the role of efflux pumps in the establishment of tolerance to biocides in gram-negative bacteria responsible for hospital-acquired infections. In particular, the activation of CepA, QacDE and QacE EPs was associated with the induction of tolerance to biocides in *Klebsiella pneumoniae* (Abuzaid et al., 2012; Ni et al., 2022). Hyperexpression of Mex-Opr efflux pump was described as the main mechanism responsible for CHX tolerance in *Pseudomonas aeruginosa* (Zheng et al., 2022). In *A. baumannii*, the tolerance to several biocides including CHX and BZK during planktonic growth was dependent on the activation of EPs belonging to RND, MFS and PACE efflux systems (Rajamohan et al., 2010a,b; Hassan et al., 2013; Tucker et al., 2014; Yoon et al., 2015; Foong et al., 2019; Migliaccio et al., 2022a; Meyer et al., 2022; Li et al., 2023). In line with this, the herein data demonstrated that the activation of AdeB and AdeJ, belonging to RND efflux system, and to a lesser extent that of AmvA and AceI EPs belonging to MSF and PACE efflux systems, respectively, was important to establish the tolerance to CHX and BZK in *A. baumannii* ATCC 19606 during biofilm formation. Furthermore, our data demonstrated that the combination of CHX and BZK at sub-MIC doses inhibited biofilm formation in *A. baumanniii* ATCC 19606 wild type and EPs mutants and that the effect was potentiated by the addition of RV. This is in agreement with previous data showing that RV is able to revert tolerance and partially restore susceptibility to CHX (Singkham-In et al., 2020) or to revert tolerance and restore susceptibility to CHX and BZK in *A. baumannii* during planktonic growth (Migliaccio et al., 2022b).

In this study, we also analyzed the mechanism responsible for the effect of RV on the tolerance to CHX and BZK during *A. baumannii* ATCC 19606 biofilm formation. Our data demonstrated that the expression of *amvA*, *adeB*, *adeJ*, *aceI* genes was upregulated during biofilm growth compared to planktonic growth and that RV regulated the expression of EPs genes during *A. baumannii* biofilm growth. In details, RV inhibited both basal and CHX-induced *adeB*, *adeJ*, *aceI* gene expression, increased *amvA*, *adeB* and *adeJ* gene expression in the presence of BZK and inhibited *amvA*, *adeB*, *adeJ*, *aceI* gene expression in the presence of CHX and BZK combination. Moreover, a positive correlation was found between *adeB* gene expression and biofilm formation in *A. baumannii* ATCC19606 treated with CHX, BZK and RV. In partial agreement with previous data on the effect of RV on the susceptibility to CHX and BZK (Singkham-In et al., 2020; Migliaccio et al., 2022b) and on the correlation between AdeABC and AdeIJK over-expression and reduced susceptibility to CHX and BZK (Meyer et al., 2022) during *A. baumannii* planktonic growth, the data shown herein indicate that the effect of RV on the susceptibility to CHX and BZK biocides during *A. baumannii* biofilm growth is mediated by the inhibition of *adeB* EP gene expression.

In accordance with previous finding on the effects of biocides combinations during *A. baumannii* planktonic growth (Migliaccio et al., 2022b), the combination of CHX and BZK was able to eradicate preformed biofilms in *A. baumannii* ATCC 19606, 4190 and 3909 strains and that RV had an additive effect on this phenomenon. Interestingly, MBEC values of CHX and BZK combination in the presence of RV are reduced by 2-to 4-fold respect to MBEC values of biocides combination in the absence of RV (Table 3), while they are identical or reduced by 4 to 8-folds reduced respect to MBC values of CHX and BZK alone, respectively (Migliaccio et al., 2022a,b). In accordance with previous data (Weiskirchen and Weiskirchen, 2016; Kim et al., 2020; Dinu et al., 2024), all the concentrations of CHX, BZK and RV utilized herein are not cytotoxic (Supplementary Figure S1). Moreover, the combinations of CHX and BZK in the presence of RV reduce MBICs and MBECs of both biocides. This is a novel finding and indicates that the combination of CHX, BZK and RV can be used for disinfectants/antiseptics formulations against *A. baumannii* or other microorganisms tolerant to biocides, growing in sessile mode. The above formulations may be employed to improve disinfectant efficacy on microbial biofilms in healthcare settings (Maillard and Centeleghe, 2023). Additional experiments will be important to analyze the effects on biofilm formation and preformed biofilms in an *in vivo* model. Also, the effects of prolonged exposure of RV, CHX and BZK, and implications for their long-term use need to be evaluated in both *in vitro* and *in vivo* models.

## 5 Conclusion

The data reported in this study demonstrated that the combination of CHX and BZK biocides in the presence of RV blocked biofilm growth and eradicated preformed biofilm in *A. baumannii* We also showed that the tolerance to CHX and BZK during biofilm growth was dependent on the activation of AdeB and AdeJ EPs, whereas the inhibitory effect of RV on biofilm growth was mediated by the inhibition of EP genes expression in *A. baumannii*.

The effects of CHX, BZK and RV on *A. baumannii* biofilms may represent a useful strategy to design novel disinfectant/ antiseptic formulations against biofilm-forming microorganisms, which are strongly tolerant to biocides. Future studies will be necessary to evaluate the clinical applicability and safety of these compounds in *in vivo* models and their compatibility in hospital disinfectant formulations.

## Data Availability

The original contributions presented in the study are included in the article/Supplementary material, further inquiries can be directed to the corresponding authors.
