# Supplementary material for: Inhibition of biofilm formation and preformed biofilm in Acinetobacter baumannii by resveratrol, chlorhexidine and benzalkonium: modulation of efflux pump activity
Source: Front Microbiol. 2024 Dec 16;15:1494772. doi: 10.3389/fmicb.2024.1494772 (PMC11684338; doi:10.3389/fmicb.2024.1494772)
Supplement: Supplementary file 1 [file Data_Sheet_1.ZIP › Figure S2.docx]

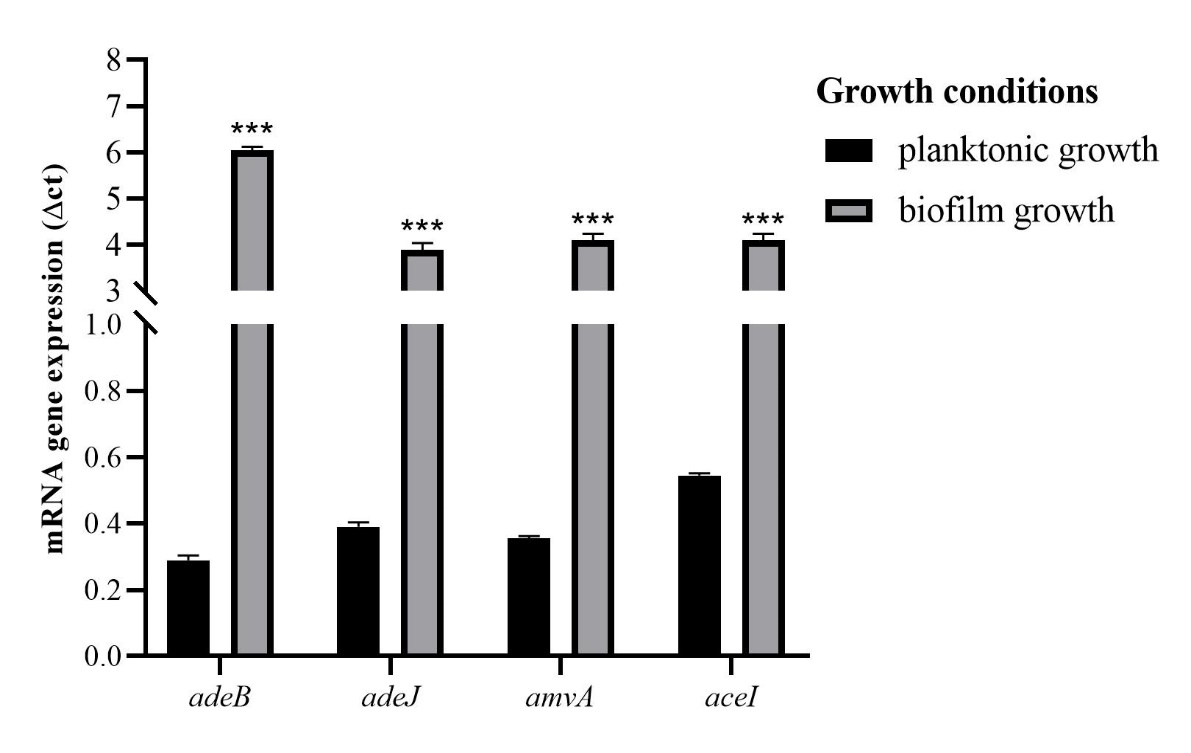


**FIGURE S2** RT-PCR of efflux pump gene expression in *A. baumannii* ATCC19606 during biofilm growth (grey columns) or planktonic growth (black columns). Assays were performed in triplicate.
