# Supplementary material for: Inhibition of biofilm formation and preformed biofilm in Acinetobacter baumannii by resveratrol, chlorhexidine and benzalkonium: modulation of efflux pump activity
Source: Front Microbiol. 2024 Dec 16;15:1494772. doi: 10.3389/fmicb.2024.1494772 (PMC11684338; doi:10.3389/fmicb.2024.1494772)
Supplement: Supplementary file 1 [file Data_Sheet_1.ZIP › Figure S3.docx]

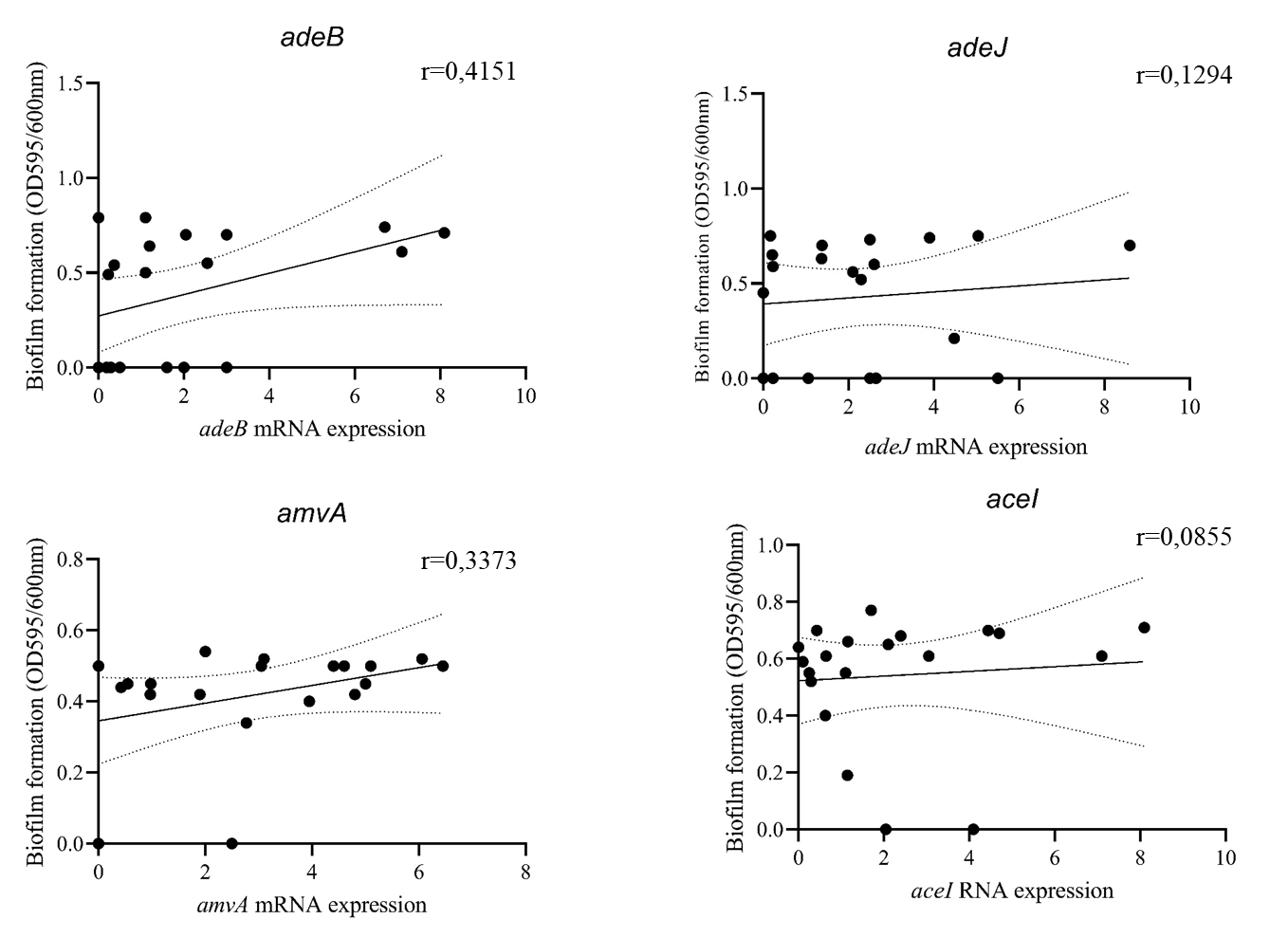


r, Pearson coefficient. All Pearson correlations were not significative and R squared ranged between 0.007312 – 0.1138.

**FIGURE S3** Pearson correlation analysis between EPs gene expression in ATCC 19606 and biofilm formation in EPs isogenic mutants treated with 32 mg/L RV in the presence or absence of CHX and/or BZK at 1/8, ¼ and ½ MICs. Assays were performed in triplicate.
