# Supplementary material for: Inhibition of biofilm formation and preformed biofilm in Acinetobacter baumannii by resveratrol, chlorhexidine and benzalkonium: modulation of efflux pump activity
Source: Front Microbiol. 2024 Dec 16;15:1494772. doi: 10.3389/fmicb.2024.1494772 (PMC11684338; doi:10.3389/fmicb.2024.1494772)
Supplement: Supplementary file 1 [file Data_Sheet_1.ZIP › Table S1.docx]

**TABLE S1** Phenotypic and genotypic characteristics of *A. baumannii* strains used in this study.

| **Strain** | **MLST Genotype** | **CHX MIC** | **BZK MIC** | **RV MIC** | **References** |
| --- | --- | --- | --- | --- | --- |
| *A. baumannii* ATCC 19606 | ST52 | 32 | 16 | >1024 | *Janssen et al 1997* |
| *A. baumannii ∆adeB* | // | 4 | 8 | >1024 | *Migliaccio et al. 2022a* |
| *A. baumannii ∆adeJ* | // | 32 | 32 | >1024 |  |
| *A. baumannii ∆amvA* | // | 16 | 16 | >1024 |  |
| *A. baumannii ∆aceI* | // | 16 | 16 | >1024 |  |
| *A. baumannii* 4190 | ST25 | 32 | 8 | >1024 | *Di Popolo et al. 2011; Zarrilli et al. 2011* |
| *A. baumannii* 3909 | ST78 | 32 | 8 | >1024 |  |

MLST, multilocus sequence typing;

MIC, Minimum inhibitory concentration;

CHX, BZK and RV MIC are expressed as mg/L.
