# Supplementary material for: Inhibition of biofilm formation and preformed biofilm in Acinetobacter baumannii by resveratrol, chlorhexidine and benzalkonium: modulation of efflux pump activity
Source: Front Microbiol. 2024 Dec 16;15:1494772. doi: 10.3389/fmicb.2024.1494772 (PMC11684338; doi:10.3389/fmicb.2024.1494772)
Supplement: Supplementary file 1 [file Data_Sheet_1.ZIP › Figure S1.docx]

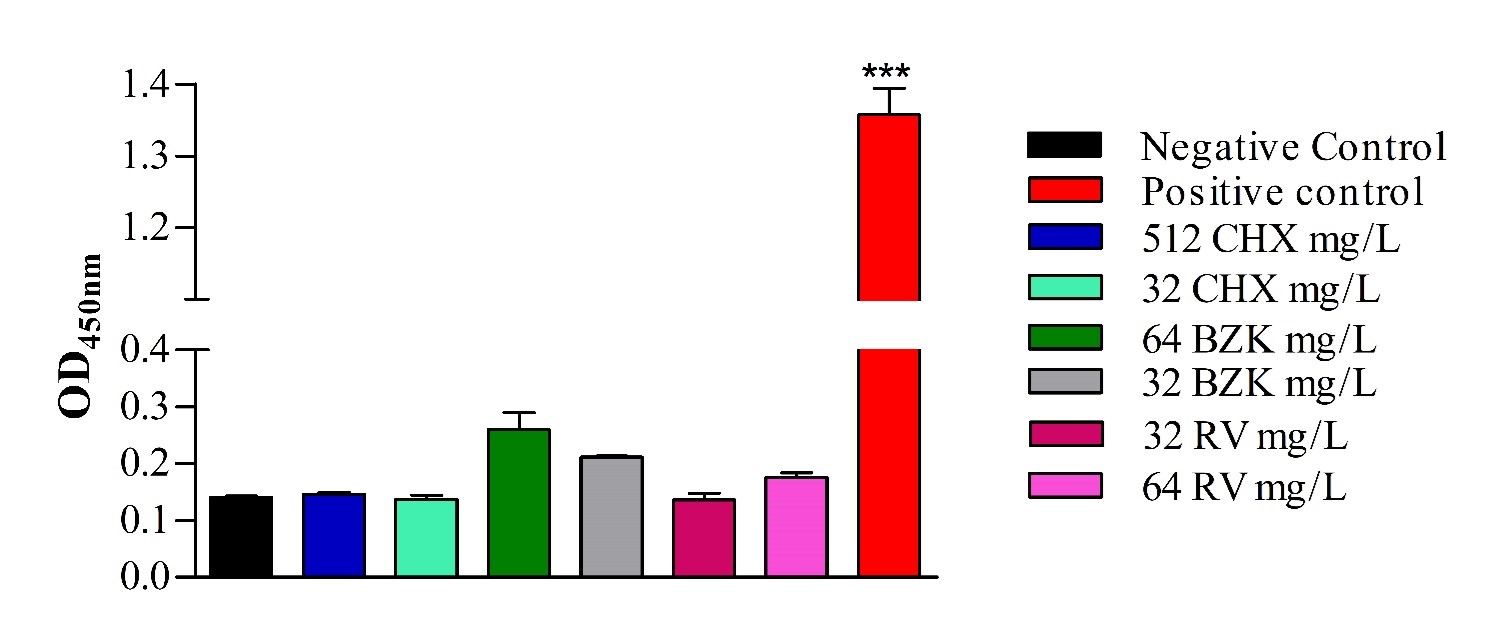


**FIGURE S1** Effects of CHX, BZK and RV on erythrocytes. The positive and negative controls were 1% (v/v) Triton X-100 and PBS solution, respectively. Data are expressed as the mean ± standard deviation (SD) of three replicates. *** p < 0.001 was calculated using ANOVA
